# Supplementary material for: Characteristics of leaf nutrient resorption efficiency in Tibetan alpine permafrost ecosystems
Source: Nat Commun. 2025 Apr 30;16:4044. doi: 10.1038/s41467-025-59289-x (PMC12041207; doi:10.1038/s41467-025-59289-x)
Supplement: Supplementary file 1 — Supplementary Information [file 41467_2025_59289_MOESM1_ESM.pdf]

Supplementary Information for

Characteristics of leaf nutrient resorption efficiency in Tibetan alpine permafrost ecosystems

Guibiao Yang[1,2], Meifeng Deng[1,2], Lulu Guo[1,2,3], Enzai Du[4,5], Zhihu Zheng[1,2,3], Yunfeng Peng[1,2], Chunbao Zhao[1,2,3], Lingli Liu[1,2,3] and Yuanhe Yang[1,2,3]*

[1]State Key Laboratory of Vegetation and Environmental Change, Institute of Botany, Chinese Academy of Sciences, Beijing 100093, China.

[2]China National Botanical Garden, Beijing 100093, China.

[3]University of Chinese Academy of Sciences, Beijing 100049, China.

[4]State Key Laboratory of Earth Surface Processes and Resource Ecology, Faculty of Geographical Science, Beijing Normal University, Beijing, 100875, China.

[5]School of Natural Resources, Faculty of Geographical Science, Beijing Normal University, Beijing, 100875, China.

[*]**Corresponding author:** Dr. Yuanhe Yang, E-mail: yhyang@ibcas.ac.cn

Supplementary Note 1: Mass-based leaf nutrient resorption efficiencies across the Tibetan alpine permafrost region.

Mass-based leaf nitrogen (N) and phosphorus (P) resorption efficiencies were calculated using eq. 1:

$$NuRE_m = (1 - Nu_{sen} / Nu_{mat} \times MLCF_c) \times 100\% \quad (1)$$

where the abbreviation, $NuRE_m$, stands for leaf nutrient resorption efficiency. Nu_{mat} and Nu_{sen} are mature and senesced leaf nutrient concentrations (g kg^{-1}), respectively. $MLCF_c$ is the community-level mass loss correction factor which is determined by weighting the mass loss correction factors of graminoids and forbs with their corresponding proportions of cover at each sampling site. Mass loss correction factors were 0.640 and 0.713 for forbs and graminoids, respectively[1,2]. Our results showed that, consistent with the concentration-based leaf nutrient resorption efficiencies, the mass-based leaf P resorption efficiency was significantly higher relative to N (82.6 ± 1.3% *vs*. 70.1 ± 1.0%; mean ± standard error; degrees of freedom (df) = 59, $P < 0.001$, cohen'd = 1.8, 95 percent confidence interval (CI) = 13.9 to 18.9), and at the upper end of global forbs and graminoids (Supplementary Fig. 7).

Supplementary Note 2: Assessing the representativeness of data obtained from three replicates.

To evaluate the representativeness of the data obtained from three duplicates, we implemented a strategy of random sampling across our study area. Specifically, we arranged ten plots as 10 replicates within an area of 50× 50 m^2 at Gangca county, Qinghai Province, China (37°18′N, 100°15′E; 3,280 m above sea level) and collected soil samples at a depth of 10 cm in each plot. Then, we measured soil organic C and total P contents, and characterized their means and standard deviations. Based on the means and standard deviations, three samples were randomly selected a total of 100 times. We observed that, for soil organic C, the average values of the three samples across the 100 iterations consistently fell within ±5% of the overall mean (Supplementary Fig. 8a). In the case of total P content, 95 out of the 100 iterations were found to be within ±5% of the mean (Supplementary Fig. 8b). These results demonstrate that alpine grasslands are relatively homogeneous on the Tibetan Plateau. In other words, three replicates can ensure the representativeness of the related measurements across these grassland ecosystems.

Supplementary Note 3: Power analyses of the independent-samples *t* tests.

To ensure the adequacy of sample size for the independent-samples *t*-tests, we conducted power analyses with the '*pwr*' package in R software 4.3.1[3]. The results showed that, for leaf N resorption efficiency, the power values were 0.96 (Type I error = 0.05) for the comparison between plants in our study region and graminoids worldwide, and 0.91 for the comparison between plants on the Tibetan Plateau and forbs worldwide. For leaf P resorption efficiency, the power values were 0.92 (Type I error = 0.05) for the comparison with graminoids worldwide, and 0.84 for the comparison with forbs worldwide (Supplementary Table 3). Given that practitioners usually consider a power value of 0.8 on the basis of the ratio of Type II (β) to Type I error (α)[3], sample sizes used in this study are adequate for these independent-samples *t* tests.

Supplementary Note 4: List of the studies included in the global dataset of N and/or P resorption efficiency.

1. Almahasheer, H. *et al.* Leaf nutrient resorption and export fluxes of Avicennia marina in the central red sea area. *Front Mar Sci* **5**, 204 (2018).

2. Alvarez-Clare, S. *et al.* A direct test of nitrogen and phosphorus limitation to net primary productivity in a lowland tropical wet forest. *Ecology* **94**, 1540-1551 (2013).

3. Aydin, I. & Uzun, F. Nitrogen and phosphorus fertilization of rangelands affects yield, forage quality and the botanical composition. *Eur J Agron* **23**, 8-14 (2005).

4. Báez, S. *et al.* Atmospheric nitrogen deposition in the northern Chihuahuan desert: temporal trends and potential consequences. *J Arid Environ* **68**, 640-651 (2007).

5. Bai, C. Study on nutrient use and stoichiometry of dominant plants in desert steppe. Master Thesis, Inner Mongolia University (2013).

6. Barger, N. N. *et al.* Nutrient limitation to primary productivity in a secondary savanna in Venezuela. *Biotropica* **34**, 493-501 (2002).

7. Bennett, L. T. *et al.* Response of a perennial grassland to nitrogen and phosphorus additions in sub-tropical, semi-arid Australia. *J Arid Environ* **48**, 289-308 (2001).

8. Bilgin, A. *et al.* Foliar N and P resorption and nutrient (N, P, C, and S) contents of *Vaccinium arctostaphylos L.* and *Vaccinium myrtillus L.* from East Black Sea region of Turkey. *Turk J Bot* **40**, 137-146 (2016).

9. Bin, Z. Effects of silicon, nitrogen and phosphorus addition on plant community structure and productivity of alpine meadow on Qinghai-Tibetan Plateau, China.

Doctoral Dissertation, Lanzhou University (2014).

10. Blanco, J. A. *et al.* Thinning affects nutrient resorption and nutrient-use efficiency in two Pinus sylvestris stands in the Pyrenees. *Ecol Appl* **19**, 682-698 (2009).

11. Bobbink, R. Effects of nutrient enrichment in Dutch chalk grassland. *J Applied Ecol* **28**, 28-41 (1991).

12. Bowman, W. D. *et al.* Constraints of nutrient availability on primary production in two alpine tundra communities. *Ecology* **74**, 2085-2097 (1993).

13. Bowman, W. D. Accumulation and use of nitrogen and phosphorus following fertilization in two alpine tundra communities. *Oikos* **70**, 261-270 (1994).

14. Bowman, W. D. *et al.* Physiological and production responses of plant growth forms to increases in limiting resources in alpine tundra: implications for differential community response to environmental change. *Oecologia* **101**, 217-227 (1995).

15. Campo, J. & Vázquez-Yanes, C. Effects of nutrient limitation on aboveground carbon dynamics during tropical dry forest regeneration in Yucatan, Mexico. *Ecosystems* **7**, 311-319 (2004).

16. Cech, P. G. *et al.* Effects of herbivory, fire and N_2-fixation on nutrient limitation in a humid African savanna. *Ecosystems* **11**, 991-1004 (2008).

17. Chen, W. Y. *et al.* The influence of different types of fertilizer application level on the Gannan desertification of alpine meadow of plant characteristics and the productive forces. *Agr Ecosyst Environ* **39**, 1899-1901 (2012).

18. Chen, W. W. *et al.* Short-term responses of foliar multi-element stoichiometry and

nutrient resorption of slash pine to N addition in subtropical China. *Chin J Applied Ecol* **28**, 1094-1102 (2017).

19. Chapin III, F. S. & Moilanen, L. Nutritional controls over nitrogen and phosphorus resorption from Alaskan birch leaves. *Ecology* **72**, 709-715 (1991).
20. Chapin III, F. S. & Shaver, G. R. Differences in growth and nutrient use among arctic plant growth forms. *Funct Ecol* **3**, 73-80 (1989).
21. Corte, G. N. *et al.* Nitrogen availability, leaf life span and nitrogen conservation mechanisms in leaves of tropical trees. *Sci Agr* **66**, 812-818 (2009).
22. Cusack, D. F. *et al.* Effects of nitrogen additions on above-and belowground carbon dynamics in two tropical forests. *Biogeochem* **104**, 203-225 (2011).
23. Davis, M. R. *et al.* 2004. The influence of N addition on nutrient content, leaf carbon isotope ratio, and productivity in a Nothofagus forest during stand development. *Can J Forest Res* **34**, 2037-2048 (2004).
24. De, K. Effect of fertilization on primary productivity and soil nutrient of alpine meadow in Three River Source Region. Doctoral dissertation, Gansu Agricultural University (2014).
25. Drenovsky, R. E. & Richards, J. H. Critical N: P values: predicting nutrient deficiencies in desert shrublands. *Plant Soil* **259**, 59-69 (2004).
26. Du, E. & Fang, J. Weak growth response to nitrogen deposition in an old-growth boreal forest. *Ecosphere* **5**, art109 (2014).
27. Fan, W. Effects of nitrogen addition on community characteristics of desert steep slope in different grazing backgrounds in Inner Mongolia. Master Thesis, Inner

Mongolia University (2018).

28. Feller, I. C. *et al.* Nitrogen *vs.* phosphorus limitation across an ecotonal gradient in a mangrove forest. *Biogeochem* **62**, 145-175 (2003).
29. Finzi, A. Decades of atmospheric deposition have not resulted in widespread phosphorus limitation or saturation of tree demand for nitrogen in southern New England. *Biogeochem* **92**, 217-229 (2009).
30. Fisher, J. B. *et al.* Nutrient limitation in rainforests and cloud forests along a 3,000-m elevation gradient in the Peruvian Andes. *Oecologia* **172**, 889-902 (2013).
31. Fonte, S. J. & Schowalter, T. D. Decomposition of greenfall vs. senescent foliage in a tropical forest ecosystem in Puerto Rico1. *Biotropica* **36**, 474-482 (2004).
32. Forey, E., Trap, J. & Aubert, M. Liming impacts Fagus sylvatica leaf traits and litter decomposition 25 years after amendment. *Forest Ecol Manag* 353, 67-76 (2015).
33. Foster, B. L. & Gross, K. L. Species richness in a successional grassland: effects of nitrogen enrichment and plant litter. *Ecology* **79**, 2593-2602 (1998).
34. Foster, N. W. & Morrison, I. K. Carbon sequestration by a jack pine stand following urea application. *Forest Ecol Manag* **169**, 45-52 (2002).
35. Gomes, A. C. S. & Luizão, F. J. Leaf and soil nutrients in a chronosequence of second-growth forest in central Amazonia: implications for restoration of abandoned lands. *Restor Ecol* **20**, 339-345 (2012).
36. Gruber, A., Oberhuber, W. & Wieser, G. Nitrogen addition and understory removal

but not soil warming increased radial growth of Pinus cembra at treeline in the Central Austrian Alps. *Front Plant Sci* **9**, 711 (2018).

37. Guevara, J. C. *et al*. N and P fertilization on rangeland production in Midwest Argentina. *J Rang Manag* **53**, 410-414 (2000).
38. Gundersen, P. 1998. Effects of enhanced nitrogen deposition in a spruce forest at Gusewell, S. Nutrient resorption of wetland graminoids is related to the type of nutrient limitation. *Funct Ecol* **19,** 344-354 (2005).
39. Gundersen, P. Effects of enhanced nitrogen deposition in a spruce forest at Klosterhede, Denmark, examined by moderate NH_4NO_3 addition. *Forest Ecol Manag* **101**, 251-268 (1998).
40. Haag, R. W. Nutrient limitations to plant production in two tundra communities. *Can J Bot* **52**, 103-116 (1974).
41. Harpole, W. S., Potts, D. L., Suding, K. N. Ecosystem responses to water and nitrogen amendment in a California grassland. *Glob Chang Biol* **13**, 2341-2348 (2007).
42. Harrington, R. A., Fownes, J. H. & Vitousek, P. M. Production and resource use efficiencies in N-and P-limited tropical forests: a comparison of responses to long-term fertilization. *Ecosystems* **4**, 646-657 (2001).
43. Helmisaari, H.S. Nutrient retranslocation in three Pinus sylvestris stands. *Forest Ecol Manag* **51**, 347-367 (1992).
44. Henry, G. H. R., Freedman, B. & Svoboda, J. Effects of fertilization on three tundra plant communities of a polar desert oasis. *Can J Bot* 64, 2502-2507 (1986).

45. Herbert, D. A. & Fownes, J. H. Phosphorus limitation of forest leaf area and net primary production on a highly weathered soil. *Biogeochem* **29**, 223-235 (1995).

46. Herrick, J. D. & Thomas, R. B. Leaf senescence and late-season net photosynthesis of sun and shade leaves of overstory sweetgum (*Liquidambar styraciflua*) grown in elevated and ambient carbon dioxide concentrations. *Tree Physiol* **23**, 109-118 (2003).

47. Hobbie, S. E. & Gough, L. Foliar and soil nutrients in tundra on glacial landscapes of contrasting ages in northern Alaska. *Oecologia* **131**, 453-462 (2002).

48. Houle, D. & Moore, J. D. Soil solution, foliar concentrations and tree growth response to 8 years of ammonium-nitrate additions in two boreal forests of Quebec, Canada. *Forest Ecol Manag* **437**, 263-271 (2019).

49. Hunt, H. W. *et al.* Nitrogen limitation of production and decomposition in prairie, mountain meadow, and pine forest. *Ecology* **69**, 1009-1016 (1988).

50. Huseyinoglu, R. *et al.* Foliar resorption of some macro-(N, P, S) and micronutrients (Fe, Zn, Cu, Mn) in *Pterocarya fraxinifolia (Poiret) Spach* forests in Turkey. *Revue D Ecologie-La Terre Et La Vie* **71**, 397-406 (2016).

51. Ibáñez, I. *et al.* Anthropogenic nitrogen deposition ameliorates the decline in tree growth caused by a drier climate. *Ecology* **99**, 411-420 (2018).

52. Jacobson, S. & Pettersson, F. Growth responses following nitrogen and NPK Mg additions to previously N-fertilized Scots pine and Norway spruce stands on mineral soils in Sweden. *Can J Forest Res* **31**, 899-909 (2001).

53. Jia, J. *et al.* Effects of water and nitrogen addition on vegetation carbon pools in a

semi-arid temperate steppe. *J Forest Res* **27**, 621-629 (2016).

54. Jiang, L. *et al.* The response of tree growth to nitrogen and phosphorus additions in a tropical montane rainforest. *Sci Total Environ* **618**, 1064-1070 (2018).
55. Keenan, R. J., Prescott, C. E. & Kimmins, J. P. Litter production and nutrient resorption in western red cedar and western hemlock forests on northern Vancouver Island, British Columbia. *Can J Forest Res* **25**, 1850-1857 (1995).
56. Kilic, D. *et al*. Foliar resorption in Quercus petraea subsp iberica and Arbutus andrachne along an elevational gradient. *Ann Forest Sci* **67**, 213 (2010).
57. Killingbeck, K. T. Can zinc influence nutrient resorption? A test with the drought-deciduous desert shrub Fouquieria splendens (ocotillo). *Plant Soil* **304**, 145-155 (2008).
58. Koide, R. T. *et al*. Effects of applications of fungicide, phosphorus and nitrogen on the structure and productivity of an annual serpentine plant community. *Funct Ecol* **2**, 335-344 (1988).
59. Kulmatiski, A. *et al*. Nitrogen and calcium additions increase forest growth in northeastern USA spruce–fir forests. *Can J Forest Res* **37**, 1574-1585 (2007).
60. Ladwig, L. M. *et al*. Above-and belowground responses to nitrogen addition in a Chihuahuan Desert grassland. *Oecologia* **169**, 177-185 (2012).
61. Lal, C. B. *et al*. Foliar demand and resource economy of nutrients in dry tropical forest species. *J Veget Sci* **12**, 5-14 (2001).
62. Li, P. *et al*. Response and mechanism of plant leaf nitrogen and phosphorus resorption efficiency to nutrient addition in an alpine meadow. *J Beijing For Univ*

46, 93-103 (2024).

63. Li, X. 2016. Effects of fertilization and clipping on productivity and species diversity in *Leymus* chinensis grassland. Doctoral dissertation, Northeast Normal University.
64. Li, X. *et al*. Leaf nutrient resorption by savanna liana species in a dry-hot valley in Yuanjiang,southwest China. *J For Environ* **44**, 260-266 (2024).
65. Li, R. *et al*. Shifted plant composition predominantly controls nitrogen addition effect on community-level leaf nutrient resorption in a boreal peatland. *Plant Soil* **494**, 321-331 (2023).
66. Liang, X. S. *et al*. The functional characters of three plants in Hulun Buir grassland responded to the addition of nitrogen and phosphorus. *Chin J Grassland* **41**, 61-67 (2019).
67. Lin, P. & Wang, W. Changes in the leaf composition, leaf mass and leaf area during leaf senescence in three species of mangroves. *Ecol Eng* **16**, 415-424 (2001).
68. Lin, Y. *et al*. Nutrient conservation strategies of a mangrove species Rhizophora stylosa under nutrient limitation. *Plant Soil* **326**, 469-479 (2010).
69. Lin, Y. & Sternberg, L. D. L. Nitrogen and phosphorus dynamics and nutrient resorption of *Rhizophora mangle* leaves in south Florida, USA. *Bull Mar Sci* **80**, 159-169 (2007).
70. Liu, X. M. *et al*. Effect of nitrogen addition of alpine grassland ecosystems degraded to different extents. *Pratacultural Science* **35**, 2773-2783 (2018).

71. Liu, Y. 2018. Effects of environmental changes on the radial growth of dominant tree species in northeast China. Master Thesis, Heilongjiang University.

72. Luo, Y. *et al*. Drought and nitrogen deposition regulate plant nutrient resorption in a typical steppe. *Agr Ecosyst Environ* **374**, 109160 (2024).

73. Lusk, C. H. & Contreras, O. Foliage area and crown nitrogen turnover in temperate rain forest juvenile trees of differing shade tolerance. *J Ecol* **87**, 973-983 (1999).

74. Ma, B. *et al*. Plant economics spectrum governs leaf nitrogen and phosphorus resorption in subtropical transitional forests. *BMC Plant Biol* **24**, 764 (2024).

75. Machado, M. R. *et al*. Nutrient retranslocation in forest species in the Brazilian Amazon. Acta Scientiarum-Agronomy, *Acta Sci Agron* **38**, 93-101 (2016).

76. Mayor, J. R., Wright, S. J. & Turner, B. L. Species-specific responses of foliar nutrients to long-term nitrogen and phosphorus additions in a lowland tropical forest. *J Ecol* **102**, 36-44 (2014).

77. McMaster, G. S., Jow, W. M. & Kummerow, J. Response of Adenostoma fasciculatum and Ceanothus greggii chaparral to nutrient additions. *J Ecol* **70**, 745-756 (1982).

78. Miller, R. E. & Tarrant, R. F. Long-term growth response of Douglas-fir to ammonium nitrate fertilizer. *Forest Sci* **29**, 127-137 (1983).

79. Mirmanto, E. *et al*. Effects of nitrogen and phosphorus fertilization in a lowland evergreen rainforest. *Philos Trans R Soc Lond B Biol Sci* **354**, 1825-1829 (1999).

80. Momen, B. *et al*. Photosynthetic and growth response of sugar maple (Acer

saccharum Marsh.) mature trees and seedlings to calcium, magnesium, and nitrogen additions in the Catskill Mountains, NY, USA. *PloS One* **10**, e0136148 (2015).

81. Negi, G.C.S. & Singh, S.P. Leaf nitrogen dynamics with particular reference to retranslocation in evergreen and deciduous tree species of Kumaun Himalaya. *Can J Forest Res* **23**, 349-357 (1993).
82. Nilsson, L. O. & Wiklund, K. Influence of nutrient and water stress on Norway spruce production in south Sweden—the role of air pollutants. *Plant Soil* **147**, 251-265 (1992).
83. Oheimb, G. V. *et al*. N:P ratio and the nature of nutrient limitation in calluna-dominated heathlands. *Ecosystems* **13**, 317-327 (2010).
84. Pan, S. *et al*. Effects of clipping and fertilizing on the relationship between functional diversity and aboveground net primary productivity in an alpine meadow. *Chin J Plant Ecol* **39**, 867-877 (2015).
85. Pandey, R. R. *et al*. Litterfall, litter decomposition and nutrient dynamics in a subtropical natural oak forest and managed plantation in northeastern India. *Forest Ecol Manag* **240**, 96-104 (2007).
86. Peng, Z. *et al*. Foliar nutrient resorption stoichiometry and microbial phosphatase catalytic efficiency together alleviate the relative phosphorus limitation in forest ecosystems. *New Phytol* **238**, 1033-1044 (2024).
87. Pérez, C.A. *et al*. Litterfall dynamics and nitrogen use efficiency in two evergreen temperate rainforests of southern Chile. *Austral Ecol* **28**, 591-600 (2003).

88. Peri, P. L. & Lasagno, R. G. Biomass, carbon and nutrient storage for dominant grasses of cold temperate steppe grasslands in southern Patagonia, Argentina. *J Arid Environ* **74**, 23-34 (2010).

89. Pugnaire, F. I. & Chapin Iii, F. S. Controls over nutrient resorption from leaves of evergreen mediterranean specie. *Ecology* **74**, 124-129 (1993).

90. Quested, H. M. *et al.* The hemiparasitic angiosperm Bartsia alpina has the potential to accelerate decomposition in sub-arctic communities. *Oecologia* **130**, 88-95 (2002).

91. Ralhan, P. K. & Singh, S. P. Dynamics of nutrients and leaf mass in central Himalayan forest trees and shrubs. *Ecology* **68**, 1974-1983 (1987).

92. Ratnam, J. *et al.* Nutrient resorption patterns of plant functional groups in a tropical savanna: variation and functional significance. *Oecologia* **157**, 141-151 (2008).

93. Ren, H. *et al.* Exacerbated nitrogen limitation ends transient stimulation of grassland productivity by increased precipitation. *Ecol Monogr* **87**, 457-469 (2017).

94. Sardans, J., Rodà, F. & Peñuelas, J. Phosphorus limitation and competitive capacities of Pinus halepensis and Quercus ilex subsp. rotundifolia on different soils. *Plant Ecol* **174**, 307 (2004).

95. Seastedt, T. R., Briggs, J. M. & Gibson, D. J. Controls of nitrogen limitation in tallgrass prairie. *Oecologia* **87**, 72-79 (1991).

96. Seastedt, T. R. & Vaccaro, L. 2001. Plant species richness, productivity, and

nitrogen and phosphorus limitations across a snowpack gradient in alpine tundra, Colorado, USA. *Arct Antarct Alp Res* **33**, 100-106 (2001).

97. Semmartin, M. & Oesterheld, M. Effects of grazing pattern and nitrogen availability on primary productivity. *Oecologia* **126**, 225-230 (2001).
98. Shen, H. *et al*. Effects of nitrogen addition on the quantitative characteristics and photosynthesis of different plant functional groups in alpine meadow of Qinghai-Tibetan Plateau. *Chin J Ecol* **38**, 1276-1284 (2019).
99. Shen Y. 2016. Effects of water and nitrogen and litter addition on *Leymus chinensis* grassland. China Agricultural University, Doctoral dissertation.
100. Sikström, U. Effects of low-dose liming and nitrogen fertilization on stemwood growth and needle properties of *Picea abies* and *Pinus sylvestris*. *For Ecol Manag* **95**, 261-274 (1997).
101. Soudzilovskaia, N. A. *et al*. 2005. Biomass production, N: P ratio and nutrient limitation in a Caucasian alpine tundra plant community. *J Veg Sci* **16**, 399-406.
102. Stegemoeller, K. A. & Chappell, H. N. Growth response of unthinned and thinned Douglas-fir stands to single and multiple applications of nitrogen. *Can J Forest Res* **20**, 343-349 (1990).
103. Su,Y. *et al*. 2019. Response of stoichiometric characteristics of nitrogen and phosphorus in leaf to nitrogen deposition in an alpine grassland of Tianshan mountains. *Arid Zone Res* **2**, 430-436 (2019).
104. Tanner, E. V. J. *et al*. Nitrogen and phosphorus fertilization of Jamaican

montane forest trees. *J Trop Ecol* **6**, 231-238 (1990).

105. Tanner, E. V. J., Kapos, V. & Franco, W. Nitorgen and phosphorus fertilization effects on Venezuelan montane forest trunk growth and litterfall. *Ecology* **73**, 78-86 (1992).

106. Teklay, T. Seasonal dynamics in the concentrations of macronutrients and organic constituents in green and senesced leaves of three agroforestry species in southern Ethiopia. *Plant Soil* **267**, 297 (2004).

107. Turner, C. L. *et al*. Soil N and plant responses to fire, topography, and supplemental N in tallgrass prairie. *Ecology* **78**, 1832-1843 (1997).

108. Venterink, H. O. *et al*. Nutrient limitation along a productivity gradient in wet meadows. *Plant Soil* **234**, 171-179 (2001).

109. Vitousek, P. M. *et al*. Nutrient limitations to plant growth during primary succession in Hawaii volcanoes national park. *Biogeochem* **23**, 197-215 (1993).

110. Wang, L. *et al*. Combined effects of soil moisture and nitrogen availability variations on grass productivity in African savannas. *Plant Soil* **328**, 95-108 (2010).

111. Wang, W., Wang, M. & Lin, P. (2003). Seasonal changes in element contents in mangrove element retranslocation during leaf senescene. Plant and Soil, 252, 187–193.

112. Wang, X. *et al*. New intrinsic ecological mechanisms of leaf nutrient resorption in temperate deciduous trees. *Plants* **13**, 1659 (2024).

113. Wen, H. Y., Wu, S. J. & Fu. H. Effects of nitrogen addition on net carbon

exchange in grassland ecosystem of Loess Plateau. *J Desert Res* **3**, 34-40 (2019).

114. Xu, X. *et al*. Nitrogen deposition and carbon sequestration in alpine meadows. *Biogeochem* **71**, 353-369 (2004).

115. Xu, W. & Wan, S. Water-and plant-mediated responses of soil respiration to topography, fire, and nitrogen fertilization in a semiarid grassland in northern China. *Soil Biol Biochem* **40**, 679-687 (2008).

116. Yan, G. *et al*. Sequestration of atmospheric CO_2 in boreal forest carbon pools in Northeastern China: Effects of nitrogen deposition. *Agr Forest Meteorol* **248**, 70-81 (2018).

117. Yang, Q., Wang,W. & Zeng, H. Effects of nitrogen addition on the plant diversity and biomass of degraded grasslands of Nei Mongol, China. *Chin J Plant Ecol* **42**, 430-441 (2019).

118. Yang, Y. Relationship between soil available phosphorus and above-ground net primary productivity and responses of soil available phosphorus to nutrient addition in *Leymus chinensis* steppe in Inner Mongolia. Doctoral dissertation, Chinese Academy of Science (2005).

119. Yasumura, Y. *et al*. Nitrogen resorption from leaves under different growth irradiance in three deciduous woody species. *Plant Ecol* **178**, 29-37 (2005).

120. Yu, L. *et al*. Responses of plant diversity and primary productivity to nutrient addition in a *Stipa baicalensis* grassland, China. *J Integr Agr* **14**, 2099-2108 (2015).

121. Yuan, Z. *et al*. Nitrogen resorption from senescing leaves in 28 plant species

in a semi-arid region of northern China. *J Arid Environ* **63**, 191-202 (2005).

122. Zhan, S. *et al*. Nitrogen enrichment alters plant N:P stoichiometry and intensifies phosphorus limitation in a steppe ecosystem. *Environ Exp Bot* **134**, 21-32 (2017).

123. Zhang, M. *et al*. Resorptions of 10 mineral elements in leaves of desert shrubs and their contrasting responses to aridity. *J Plant Ecol* **12**, 358-366 (2019).

124. Zhang, S. B. *et al*. Leaf nitrogen and phosphorus resorption efficiencies are related to drought resistance across woody species in a Chinese savanna. *Tree physiol* **44**, tpad149 (2023).

125. Zhang, S. *et al*. Intra-annual dynamics of xylem growth in Pinus massoniana submitted to an experimental nitrogen addition in Central China. *Tree Physiol* **37**, 1546-1553 (2017).

126. Zhang, T. *et al*. (2022) Seasonal grazing alters nutrient resorption and conservation, and affects spring growth of *Stipa grandis*. *J Plant Ecol* 16, I rtac083.

127. Zhao, Q. *et al*. Different responses of foliar nutrient resorption efficiency in two dominant species to grazing in the desert steppe. *Sci Rep 14*, 4090 (2024).

128. Zhao, Y. *et al*. Community composition, structure and productivity in response to nitrogen and phosphorus additions in a temperate meadow. *Sci Total Environ* **654**, 863-871 (2019).

129. Zheng, H. *et al*. Effects of fertilizer on plant diversity and productivity of desertified alpine grassland at Maqu, Gansu. *Acta Prataculturae Sinica* **16**, 34-39

(2007).

130. Zheng, W. *et al*. Effect of nitrogen addition on tree growth in subtropical *Cinnamomum camphora* forest. *J Cent South Univ* **33**, 35-37 (2013).

131. Zhi, K. *et al*. Responses of nitrogen and phosphorus resorption of understory plants to microscale soil nutrient heterogeneity in Chinese fir plantation. *Chin J Appl Ecol* **34**, 1187-1193 (2023).

132. Zhou, X. *et al*. The effect of fertilization on community assembly and production in alpine meadow community. Doctoral dissertation, Lanzhou University (2016).

133. Zhu, F. *et al*. Nutrient limitation in three lowland tropical forests in southern china receiving high nitrogen deposition: insights from fine root responses to nutrient additions. *Plos One* **8**, e8266 (2013).

134. Zotz, G. The resorption of phosphorus is greater than that of nitrogen in senescing leaves of vascular epiphytes from lowland Panama. *J Trop Ecol* **20**, 693-696 (2004).

Supplementary Table 1. **Characteristics of nutrient stoichiometry, nutrient resorption and soil nutrient mineralization across the Tibetan alpine permafrost region.**

Parameters	Abbreviation	Unit	Mean ± SE
**Nutrient stoichiometry**			
Mature leaf N content	N_{mat}	g kg^{-1}	18.9 ± 0.5
Mature leaf P content	P_{mat}	g kg^{-1}	1.4 ± 0.06
Mature leaf N and P content ratio	N_{mat}/P_{mat}	/	14.1 ± 0.4
Senesced leaf N content	N_{sen}	g kg^{-1}	7.7 ± 0.3
Senesced leaf P content	P_{sen}	g kg^{-1}	0.4 ± 0.03
Senesced leaf N and P content ratio	N_{sen}/P_{sen}		25.7 ± 1.4
**Nutrient resorption**			
N resorption efficiency	NRE	%	58.7 ± 1.5
P resorption efficiency	PRE	%	75.1 ± 1.8
N and P resorption efficiency ratio	NRE/PRE	/	0.8 ± 0.02
Resorbed N	NR	g kg^{-1}	11.5 ± 0.5
Resorbed P	PR	g kg^{-1}	1.1 ± 0.05
Resorbed N and P ratio	RN/RP	/	10.9 ± 0.3
**Soil nutrient mineralization**			
N mineralization rate	NMR	ng cm^{-2} d^{-2}	232.5 ± 15.5
P mineralization rate	PMR	ng cm^{-2} d^{-2}	0.8 ± 0.07
N and P mineralization ratio	NMR/PMR	/	356.7 ± 47.7

All data are reported as means ± SE (*n* = 30).

Supplementary Table 2. Environmental or vegetation characteristics at 30 sampling sites on the Tibetan Plateau.

N	Lat (°N)	Long (°E)	Altitude (m)	pH	SOC (g kg^{-1})	TN (g kg^{-1})	IN (mg kg^{-1})	IP (mg kg^{-1})	Grassland types
1	34.5	99.2	4321	7.6	99.0	8.0	35.0	11.1	SM
2	34.1	99.3	4405	6.3	130.9	9.6	45.0	12.0	AM
3	34.8	99.0	4633	7.4	103.6	8.6	44.2	10.6	SM
4	35.1	98.7	4419	7.2	94.1	7.8	32.1	13.9	AM
5	34.8	98.4	4219	9.1	9.7	0.9	3.7	7.2	AS
6	34.6	98.0	4181	8.2	51.5	4.1	30.6	11.4	AS
7	34.2	97.8	4576	6.9	138.8	10.6	101.9	22.7	AM
8	33.9	97.3	4508	7.6	33.1	2.6	18.1	15.8	AM
9	34.2	96.0	4653	7.5	115.6	8.6	122.7	8.5	AM
10	33.7	97.1	4437	6.8	195.9	14.6	145.2	11.7	AM
11	34.9	94.7	4396	8.0	113.8	8.3	96.1	16.1	SM
12	32.8	95.0	4498	7.1	155.2	11.3	72.0	27.3	SM
13	32.9	94.3	4768	7.4	117.1	8.9	42.7	21.5	AM
14	32.5	93.7	4746	7.4	96.7	7.9	44.6	26.5	AM
15	31.9	93.1	4439	6.9	123.3	8.9	37.6	23.8	AM
16	31.3	91.9	4497	7.8	127.9	9.2	92.2	22.3	SM
17	31.5	92.2	4581	7.6	115.6	8.5	76.8	22.3	SM
18	31.7	91.8	4620	7.5	162.6	12.5	84.2	31.1	SM
19	32.6	91.9	5014	8.7	5.7	0.5	10.4	8.1	AM
20	33.1	91.9	4886	8.5	23.0	1.8	44.2	16.2	AS
21	34.1	92.3	4725	8.8	22.7	1.7	18.5	5.7	AM
22	34.8	92.9	4628	8.8	14.3	1.2	16.6	5.7	AM
23	35.1	94.2	4406	9.5	10.4	1.0	14.6	6.5	SM
24	32.2	91.7	4806	8.8	8.0	0.9	12.3	8.4	AS
25	31.8	92.6	4689	6.9	116.4	8.9	102.1	12.9	AS
26	37.5	100.3	3820	8.1	82.8	7.1	29.1	11.6	SM
27	38.7	99.3	3443	6.5	171.5	14.1	107.0	10.4	SM
28	37.8	101.1	3600	8.5	50.6	4.4	30.9	12.6	SM
29	38.0	100.8	3279	6.4	222.0	17.1	151.6	9.4	AM
30	37.7	100.8	3618	8.4	15.8	14.8	17.1	9.0	SM

Notes: N represents sampling site number; AS, alpine steppe; AM, alpine meadow; SM, swamp meadow; Lat, latitude; Long, longitude; SOC, soil organic carbon; TN, total nitrogen; IN, inorganic nitrogen; IP, inorganic phosphorus.

Supplementary Table 3. Power analyses of the independent-samples *t* tests for leaf nutrient resorption efficiency between Tibetan alpine grasslands and global plants from graminoid, forb or all growth types.

Growth type	Nutrient resorption	Sample size from Tibetan alpine grasslands	Sample size from global datasets	α value (type I error)	Cohen's d	Power value
Graminoid	N resorption	30	83	0.05	0.15	0.96
	P resorption	30	65	0.05	0.95	0.92
Forb	N resorption	30	43	0.05	0.35	0.91
	P resorption	30	27	0.05	1.32	0.84
All	N resorption	30	998	0.05	0.87	0.99
	P resorption	30	913	0.05	1.38	0.99

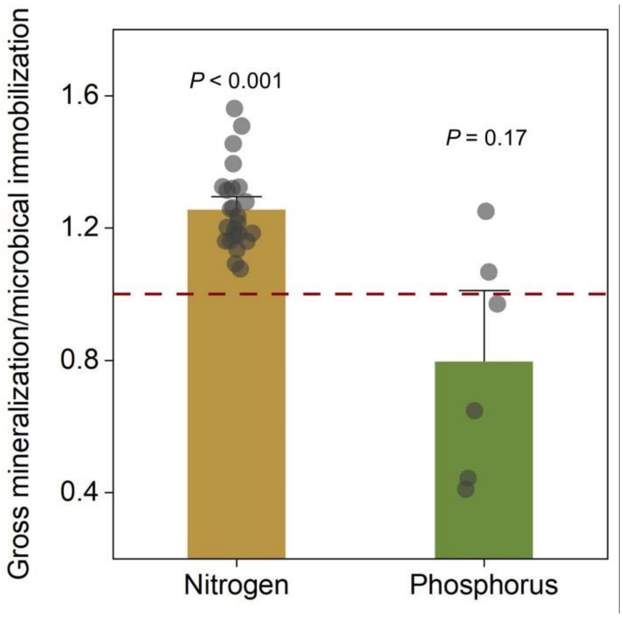

Supplementary Figure 1. Ratio of soil gross nutrient mineralization to microbial nutrient immobilization across the Tibetan alpine permafrost region. The transformation rates of N and P are reanalyzed from Mao et al.[4] and Ziliang Li (unpublished data), and determined using the ^{15}N tracer technique[5] and the ^{33}P tracer technique[6], respectively. Red line indicates the value 1. Data are represented as the means ± SE. Significant differences from 1 are determined with independent-samples *t* tests (two-sided; unadjusted $P < 0.05$); ns, insignificant difference. Sample sizes are 25 for the transformation rates of N and 6 for P.

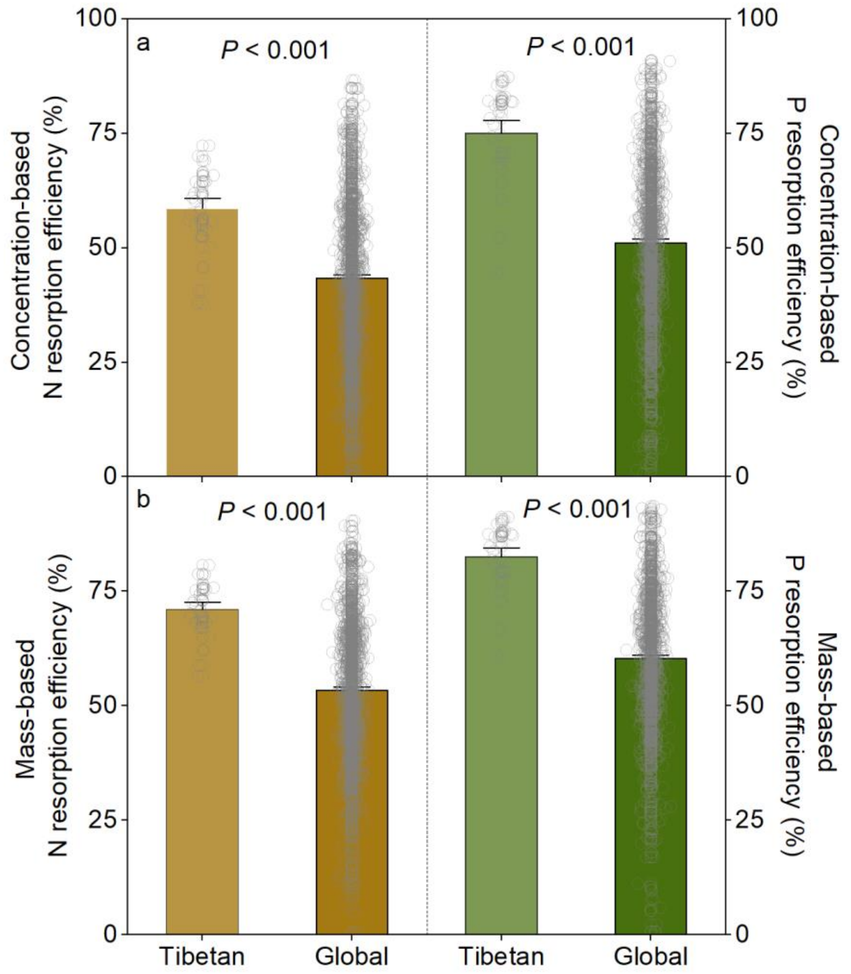

Supplementary Figure 2. Comparison of leaf nutrient resorption efficiencies between Tibetan alpine grasslands and global terrestrial ecosystems. a Assessing the differences in concentration-based leaf nutrient resorption efficiencies between them. **b** Comparative analysis of mass-based leaf nutrient resorption efficiencies between the two groups. The global database was compiled from the published studies (see details in Supplementary Note 4). Data are represented as the means ± SE (standard error). These comparisons were analyzed based on dependent-samples *t* tests (two-sided, unadjusted $P < 0.001$). There are 30 observations of leaf nutrient resorption efficiencies from Tibetan alpine grasslands, 998 global observations of leaf N resorption efficiencies and 913 global observations of P.

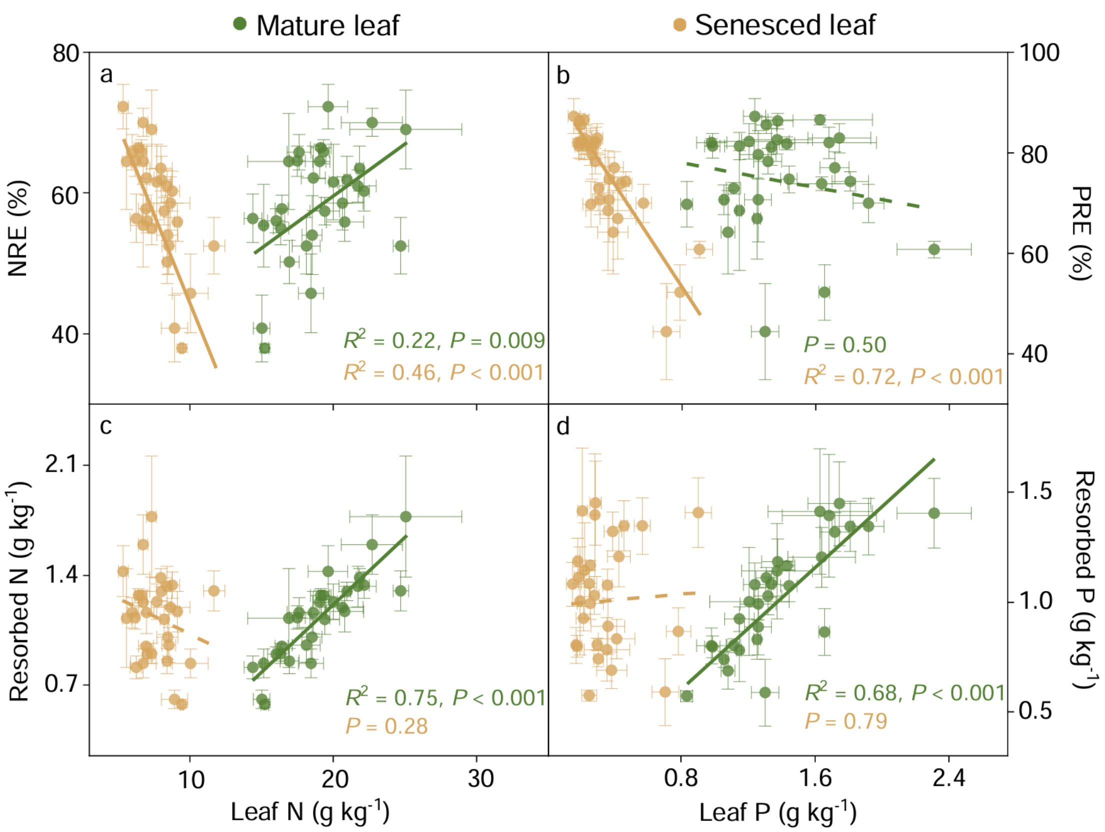

Supplementary Figure 3. Relationships of plant nutrient resorption effciency with nutrient concerntration in the mature and senesced leaves across Tibetan alpine grasslands. a Leaf N resorption effciency with N concentrations in the mature and seneced leaves. **b** Leaf N resorption effciency with P concentrations in the mature and senesced leaves. **c** Resorbed N with N concentrations in the mature and seneced leaves. **d** Resorbed P with P concentrations in the mature and seneced leaves. Significant relationships are shown using solid lines, and non-significant ones are dashed lines. Orange and green circles represent data points in the mature and senesced leaves, respectively. Error bars denote SE of mean at each site (n = 3). Statistics (R^2 and P value) are shown for the linear mixed-effects models with two-sided t tests (unadjusted $P < 0.05$).

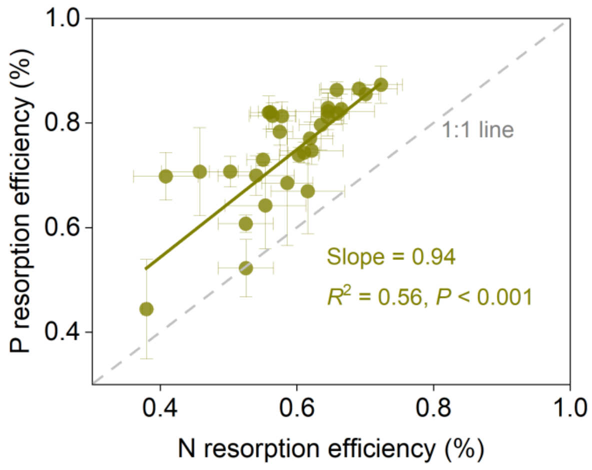

Supplementary Figure 4. Relationship between leaf P and N resorption efficiency across Tibetan alpine grasslands. A significant relationship is shown by a solid line. Error bars denote SE of mean at each site (n = 3). Statistics (slope, R^2 and P value) are shown for the linear mixed-effects model with two-sided t test (unadjusted $P < 0.05$). The grey dashed line is the 1:1 line.

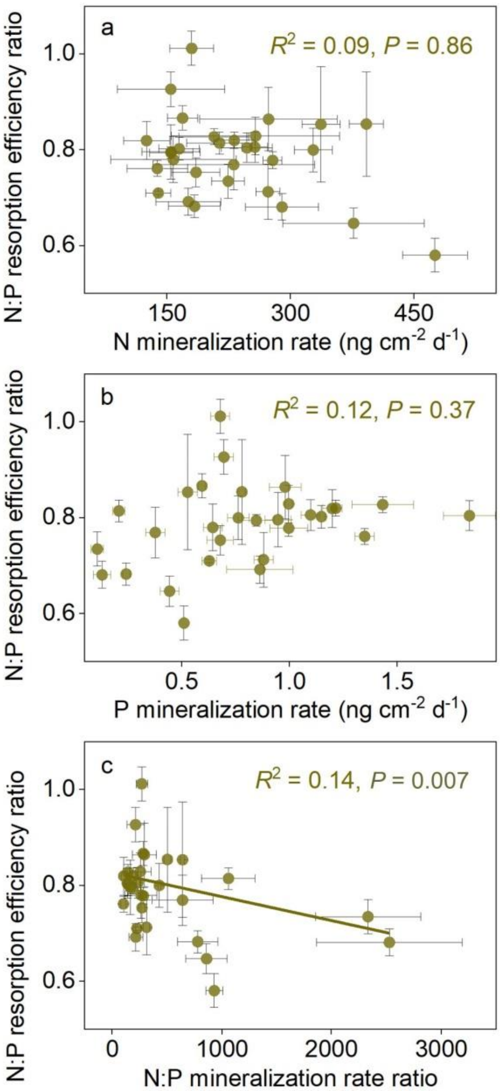

Supplementary Figure 5. Relationships between leaf N:P resorption efficiency and soil nutrient supply across Tibetan alpine grasslands. a Leaf N to P resorption efficiency ratio with topsoil N mineralization rate. **b** Leaf N to P resorption efficiency ratio with topsoil N mineralization rate. **c** Leaf N to P resorption efficiency ratio with topsoil N to P mineralization rate ratio. A significant relationship is shown by a solid line. Error bars denote SE of mean at each site (*n* = 3). Statistics (R^2 and *P* value) are shown for the generalized linear mixed-effects models with two-sided *t* test (unadjusted $P < 0.05$).

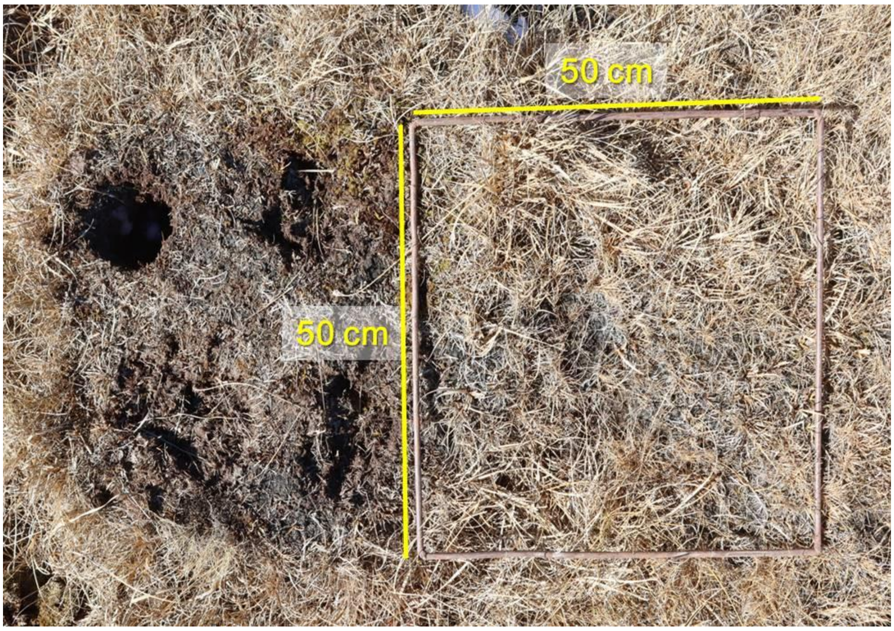

Supplementary Figure 6. Example of quadrats sampled during the peak growing season and the wilting period, respectively. The area to the left of the metal frame was sampled during the peak growing season, while the quadrat within the frame was sampled during the wilting period. Photograph by Guibiao Yang at Qilian county, Qinghai Province, China (37°25′N, 99°38′E; 3,216 m above sea level).

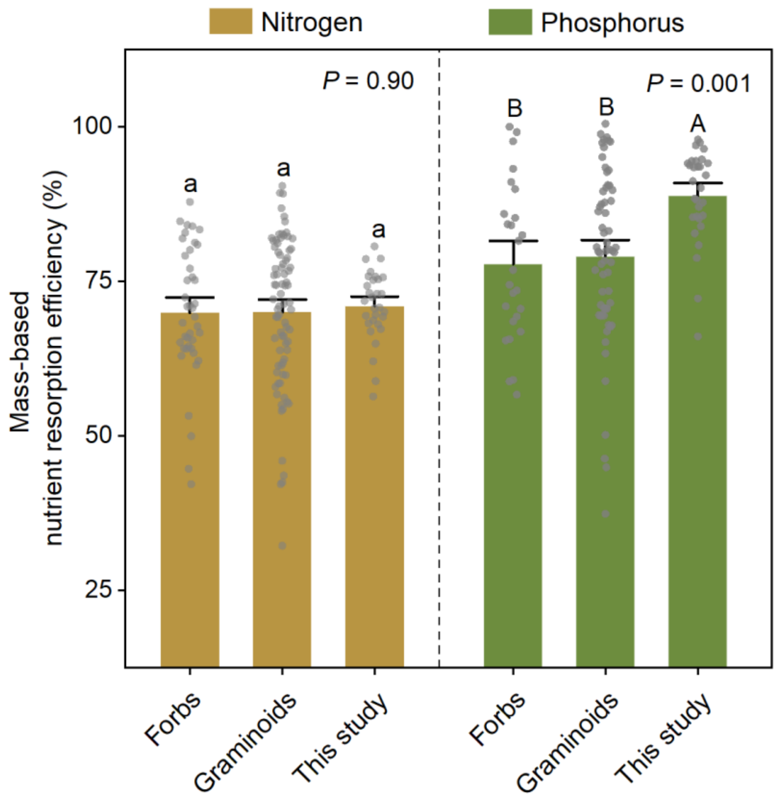

Supplementary Figure 7. Comparison of mass-based leaf nutrient resorption efficiencies among Tibetan alpine grasslands, global forbs and graminoids. The global database was compiled from the published studies (see details in Supplementary Note 4). Data are represented as means ± SE. Different letters (independent-samples *t* tests using two-side, $P < 0.05$, lowercase letters for N and capital letters for P) represent significant differences.

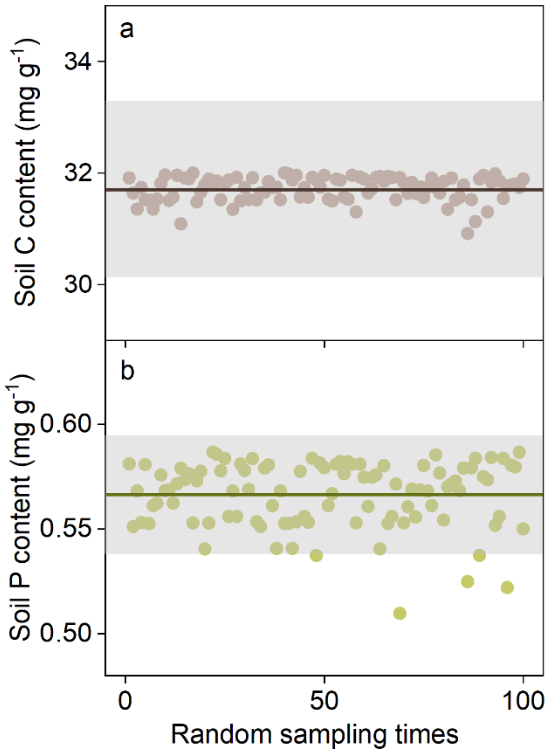

Supplementary Figure 8. Means of soil properties from three samples across 100 iterations. **a** Soil organic C, **b** Total P content. These data were measured using topsoil samples at a depth of 10 cm in each of ten plots within an area of 50× 50 m^2 at Gangca county, Qinghai Province, China (37°18′N, 100°15′E; 3,280 m above sea level). The black lines and shading represent the means and positive and negative 5% intervals, respectively.

References

1. Vergutz, L. *et al.* Global resorption efficiencies and concentrations of carbon and nutrients in leaves of terrestrial plants. *Ecoll Monogr* **82**, 205-220 (2012).
2. Du, E. *et al.* Global patterns of terrestrial nitrogen and phosphorus limitation. *Nat Geosci* **13**, 221-226 (2020).
3. Cohen, J. *Statistical Power Analysis for the Behavioral Sciences* 2nd ed. (Routledge Press, New York, 1988).
4. Mao, C. *et al.* Permafrost nitrogen status and its determinants on the Tibetan Plateau. *Global Change Biol* **26**, 5290-5302 (2020).
5. Hart, S.C., Stark, J.M., Davidson, E.A. & Firestone, M.K. *Methods of soil analysis: Nitrogen mineralization, immobilization, and nitrification.* Chapter 42. (American Society of Agronomy, Soil Science Society of America, 1994).
6. Wanek, W. *et al.* A novel isotope pool dilution approach to quantify gross rates of key abiotic and biological processes in the soil phosphorus cycle. *Biogeosciences* **16**, 3047–3068 (2019).
