# Supplementary material for: Characteristics of leaf nutrient resorption efficiency in Tibetan alpine permafrost ecosystems
Source: Nat Commun. 2025 Apr 30;16:4044. doi: 10.1038/s41467-025-59289-x (PMC12041207; doi:10.1038/s41467-025-59289-x)
Supplement: Supplementary file 3 — Reporting Summary [file 41467_2025_59289_MOESM3_ESM.pdf]

nature portfolio

Corresponding author(s): Yuanhe Yang

Last updated by author(s): Feb 18, 2025

Reporting Summary

Nature Portfolio wishes to improve the reproducibility of the work that we publish. This form provides structure for consistency and transparency in reporting. For further information on Nature Portfolio policies, see our Editorial Policies and the Editorial Policy Checklist.

Statistics

For all statistical analyses, confirm that the following items are present in the figure legend, table legend, main text, or Methods section.

n/a	Confirmed	
☐	☒	The exact sample size (*n*) for each experimental group/condition, given as a discrete number and unit of measurement
☐	☒	A statement on whether measurements were taken from distinct samples or whether the same sample was measured repeatedly
☐	☒	The statistical test(s) used AND whether they are one- or two-sided<br>*Only common tests should be described solely by name; describe more complex techniques in the Methods section.*
☐	☒	A description of all covariates tested
☐	☒	A description of any assumptions or corrections, such as tests of normality and adjustment for multiple comparisons
☐	☒	A full description of the statistical parameters including central tendency (e.g. means) or other basic estimates (e.g. regression coefficient) AND variation (e.g. standard deviation) or associated estimates of uncertainty (e.g. confidence intervals)
☐	☒	For null hypothesis testing, the test statistic (e.g. *F*, *t*, *r*) with confidence intervals, effect sizes, degrees of freedom and *P* value noted<br>*Give P values as exact values whenever suitable.*
☒	☐	For Bayesian analysis, information on the choice of priors and Markov chain Monte Carlo settings
☒	☐	For hierarchical and complex designs, identification of the appropriate level for tests and full reporting of outcomes
☐	☒	Estimates of effect sizes (e.g. Cohen's *d*, Pearson's *r*), indicating how they were calculated

Our web collection on statistics for biologists contains articles on many of the points above.

Software and code

Policy information about availability of computer code

Data collection	No software was used in this study for data collection.
Data analysis	All statistical analyses were performed using the software package R v.4.3.1. Details were reported in the Methods section.

For manuscripts utilizing custom algorithms or software that are central to the research but not yet described in published literature, software must be made available to editors and reviewers. We strongly encourage code deposition in a community repository (e.g. GitHub). See the Nature Portfolio guidelines for submitting code & software for further information.

Data

Policy information about availability of data

All manuscripts must include a data availability statement. This statement should provide the following information, where applicable:

- Accession codes, unique identifiers, or web links for publicly available datasets
- A description of any restrictions on data availability
- For clinical datasets or third party data, please ensure that the statement adheres to our policy

The data generated in this study have been deposited in the Figshare data repository (https://doi.org/10.6084/m9.figshare.28103306.v1). The previously published datasets can be obtained from Supplementary Information or corresponding author upon request.

Research involving human participants, their data, or biological material

Policy information about studies with human participants or human data. See also policy information about sex, gender (identity/presentation), and sexual orientation and race, ethnicity and racism.

Reporting on sex and gender	This study does not involve sex and gender.
Reporting on race, ethnicity, or other socially relevant groupings	This study does not involve population.
Population characteristics	This study does not involve population.
Recruitment	This study does not involve population.
Ethics oversight	This study does not involve human research, and there is no human research related organization involved.

Note that full information on the approval of the study protocol must also be provided in the manuscript.

Field-specific reporting

Please select the one below that is the best fit for your research. If you are not sure, read the appropriate sections before making your selection.

☐ Life sciences ☐ Behavioural & social sciences ☒ Ecological, evolutionary & environmental sciences

For a reference copy of the document with all sections, see nature.com/documents/nr-reporting-summary-flat.pdf

Ecological, evolutionary & environmental sciences study design

All studies must disclose on these points even when the disclosure is negative.

Study description	This study provided the unique characteristics of plant nutrient resorption in Tibetan alpine permafrost ecosystems.
Research sample	30 sampling sites, located in the representative permafrost regions, were selected along a 1,100 km transect on the Tibetan Plateau. We sampled aboveground vegetation at 30 sites during the peak growing season and wilting period in 2021, respectively.
Sampling strategy	Sampling strategy was adopted to maximize the potential bias caused by varying plant characteristics between the two periods so as to well characterize leaf nutrient resorption efficiency. Specifically, at each site, three individual quadrats (50 × 50 cm2) were established as replicates along the diagonal line of a 10 × 10 m2 plot where vegetation was characterized. Within each quadrat, all living (mature) and recently senesced (naturally), but still attached, leaves were sampled during the peak growing season and the wilting period, respectively. Field sampling campaigns at each site at the two times were carried out by the same participants, and the senesced leaf quadrats were located adjacent to mature leaf quadrats.
Data collection	Leaf nutrient resorption efficiency was calculated based on mature and senesced leaf nutrient concentrations. Specifically, community-level mature and senesced leaf N concentrations were determined by an elemental analyzer (Vario EL III, Elementar, Germany). Leaf P concentrations were measured with a spectrophotometer (ICAP6300, Thermo Fisher Scientific, Waltham, MA, USA) after a microwave-assisted digestion with H2SO4 and H2O2 at 380°C for ~3 h. In addition, to explore the potential relationship between plant nutrient resorption and soil nutrient supply, we measured in situ topsoil N and P mineralization rates with field incubation of intact soil cores at 30 sites across the Tibetan permafrost region. The detailed data collection procedures were described in the Methods section. Guibiao Yang, Zhihu Zheng and Chunbao Zhao were present during data collection.
Timing and spatial scale	Given that leaf nutrient resorption efficiency was calculated based on mature and senesced leaf nutrient concentrations, field campaigns were thus carried out during the peak growing season from mid-July to mid-August and in the wilting period from early to late October in 2021. Sampling was performed along a 1,100 km permafrost transect (Latitude: 31-39oN, longitude: 91-101oE). Detailed location is available in the supplementary Table 2.
Data exclusions	No data were excluded from the analyses.
Reproducibility	We ensured the reproducibility of this study through comprehensive efforts, such as an adequate sample size and exceptionally detailed methods section in the main text.
Randomization	We collected 30 sampling sites evenly located in three representative permafrost regions on the Tibetan Plateau. At each site, the sampling was taken at three locations. This strategy increases the area of the sampling and the randomization factors of any sample, which improves the reliability of the generated dataset.
Blinding	Not relevant because this was an observational study.

Did the study involve field work? ☒ Yes ☐ No

Field work, collection and transport

Field conditions	Field conditions are harsh in this study region, such as low temperature, oxygen limitation, and traffic inconvenience. Please see "Study area" in the Method section and Supplementary Table 2 for details.
Location	Latitude: 31-39oN, longitude: 91-101oE. Elevation: 3279-5014m. The 30 sites are located in three representative permafrost regions (10 sites in the Madoi section on the eastern plateau, 15 sites in the Budongquan-Nagqu-Zadoi section, and 5 sites in the Qilian section on the northeastern plateau in the central part of the plateau).
Access & import/export	The access to Three-River-Source National Park is granted by Three-River-Source National Park Management Bureau (TNPMB) during the sampling period from May to November. Sampling collection and transport were conducted under the authorization of the TNPMB.
Disturbance	Plant nutrient resorption may continue beyond the sampling periods, which can affect leaf nutrient resorption efficiency due to any potential seasonal variations. To minimize this disturbance, the two sampling times approximately coincide with the peak and end of plant growth on the Tibetan Plateau.

Reporting for specific materials, systems and methods

We require information from authors about some types of materials, experimental systems and methods used in many studies. Here, indicate whether each material, system or method listed is relevant to your study. If you are not sure if a list item applies to your research, read the appropriate section before selecting a response.

Materials & experimental systems

n/a	Involved in the study
☒	☐ Antibodies
☒	☐ Eukaryotic cell lines
☒	☐ Palaeontology and archaeology
☒	☐ Animals and other organisms
☒	☐ Clinical data
☒	☐ Dual use research of concern
☒	☐ Plants

Methods

n/a	Involved in the study
☒	☐ ChIP-seq
☒	☐ Flow cytometry
☒	☐ MRI-based neuroimaging

Plants

Seed stocks	This study does not involve seed stocks.
Novel plant genotypes	This study does not involve novel plant genotypes.
Authentication	This study does not involve authentication.
